# Cai’s gynecology cyclical therapy with stasis-clearing and meridian-warming method for primary dysmenorrhea: a randomized controlled trial

**DOI:** 10.3389/fendo.2026.1752657

**Published:** 2026-05-14

**Authors:** Bowen Xu, Mengfei Zhuang, Xiaomin Wang, Tingting Zhang

**Affiliations:** 1 Department of Gynecology, Yueyang Hospital of Integrated Traditional Chinese and Western Medicine, Shanghai University of Traditional Chinese Medicine, Shanghai, China; 2 Department of Traditional Chinese Medicine, Shanghai East Hospital, Tongji University School of Medicine, Shanghai, China; 3 Department of Traumatology, Shanghai East Hospital, Tongji University School of Medicine, Shanghai, China

**Keywords:** Cai’s gynecology cyclical therapy, experience of renowned traditional Chinese medicine practitioners, primary dysmenorrhea, stasis-clearing and meridian-warming therapy, traditional Chinese medicine

## Abstract

**Background:**

First-line treatment with non-steroidal anti-inflammatory drugs like ibuprofen for primary dysmenorrhea is effective for short-term pain relief but is often associated with a high recurrence of pain upon discontinuation. Traditional Chinese Medicine (TCM) offers a holistic approach aimed at regulating the body’s fundamental balance, yet high-quality evidence from randomized controlled trials (RCTs) comparing comprehensive TCM protocols to standard care is needed.

**Objective:**

To evaluate the clinical efficacy of an integrated TCM protocol—Cai’s Gynecology Cyclical Therapy combined with the Stasis-Clearing and Meridian-Warming Method—for primary dysmenorrhea, and to compare its with ibuprofen.

**Methods:**

In this RCT, 80 patients with primary dysmenorrhea of the Qi stagnation and blood stasis type were recruited at Shanghai East Hospital from February to July 2025. Participants were randomly assigned to either the TCM group (n=40) or the ibuprofen group (n=40). The TCM group received herbal treatment tailored to the menstrual cycle: Chaihushugan San combined with Siwu Decoction during the post- and inter-menstrual phases for root-cause regulation, and the Stasis-Clearing and Meridian-Warming Formula during the pre-menstrual and menstrual phases for symptom relief. The ibuprofen group received oral ibuprofen sustained-release capsules (300 mg twice daily for 2 days during menstruation, total daily dose 600 mg). The treatment spanned three menstrual cycles. Primary outcomes included TCM syndrome scores, visual analog scale (VAS) scores for pain, and recurrence rates during follow-up.

**Results:**

The total effective rate was 97.5% in both groups after three cycles. Both groups showed significant reductions in TCM syndrome and VAS scores (P<0.01). However, the TCM group showed progressive improvement from cycle 1 to cycle 3 (P<0.01), while the ibuprofen group did not (P = 0.844 for syndrome scores; P = 0.795 for VAS). While ibuprofen provided superior pain relief after the first cycle (P<0.01), the two groups were comparable by cycle 3 (P = 0.083 for syndrome scores; P = 0.059 for VAS). Crucially, the recurrence rate in the TCM group (12.8%) was significantly lower than that in the ibuprofen group (89.7%).

**Conclusion:**

While ibuprofen offers faster short-term relief, the integrated Cai’s Gynecology Cyclical Therapy achieves comparable efficacy after three cycles and is significantly more effective at preventing recurrence.

**Clinical trial registration:**

https://itmctr.ccebtcm.org.cn/mgt/project/view/7448163107044166727, identifier ITMCTR2025000425.

## 1 Introduction

Dysmenorrhea is one of the most prevalent gynecological symptoms, refers to lower abdominal pain and distension occurring before, during, or after menstruation, accompanied by lumbar soreness or other discomfort that significantly impairs quality of life. It is classified into primary dysmenorrhea (without organic pelvic pathology) and secondary dysmenorrhea (caused by organic diseases such as endometriosis or adenomyosis) (1). Epidemiological studies report an incidence rate of 45-97%, primarily associated with elevated local prostaglandin levels, exerting substantial impacts on women’s lives (2). Conventional first-line treatment primarily involves non-steroidal anti-inflammatory drugs (NSAIDs) like ibuprofen, which inhibit prostaglandin synthesis to provide effective short-term analgesia. However, a significant limitation is the high recurrence of pain upon drug discontinuation, as NSAIDs do not address the underlying pathophysiology, leading to a cycle of symptomatic relief without long-term resolution (3, 4). This gap highlights the need for treatment strategies that offer sustained benefits beyond the immediate menstrual period.

Traditional Chinese Medicine (TCM) has gained increasing international recognition for its holistic approach to manage menstrual disorders, with growing evidence from clinical studies and systematic reviews supporting its efficacy and safety (5, 6). Cai’s Gynecology is a renowned school of Traditional Chinese Medicine originating from Shanghai. Its core academic principle involves tailoring treatments to the four distinct phases of the menstrual cycle (menstrual, post-menstrual, inter-menstrual, and pre-menstrual) to regulate the body’s fundamental systems. The seventh-generation successor, Professor Cai Xiaosun, established the theoretical framework for this cyclical therapy (7). Building upon this foundation, the eighth-generation heir, Professor Zhang Tingting, developed the Stasis-Clearing and Meridian-Warming Method, an effective approach for managing dysmenorrhea and chronic pelvic pain.

## 2 Materials and methods

### 2.1 Diagnostic criteria

#### 2.1.1 Western medical diagnostic criteria

The Western medical diagnostic criteria for this study were established based on the National Health Commission’s 14th Five-Year Plan textbook (10th edition), Obstetrics and Gynecology, edited by Kong Beihua et al. (8).

Primary dysmenorrhoea: refers to lower abdominal pain, heaviness, or discomfort before, during, or after menstruation, often accompanied by lower back pain or other discomfort. In cases where symptoms are particularly severe, there is the potential for significant interference with day-to-day living and professional activities. Dysmenorrhoea is categorized into primary and secondary types. Primary dysmenorrhoea is idiopathic and cannot be explained by pelvic diseases, accounting for over 90% of dysmenorrhoea cases.

#### 2.1.2 Traditional Chinese medicine diagnostic criteria

TCM diagnosis was conducted based on the national higher education textbook, ‘Traditional Chinese Gynecology’ (9) edited by Feng Xiaoling, and the ‘Traditional Chinese Medicine Diagnosis and Treatment Standards: Clinical Guidelines for Dysmenorrhoea (2022)’ (10). Select the Qi stagnation and blood stasis type among them.

Main symptoms: Lower abdominal pain and distension during menstruation or before and after menstruation, with aversion to pressure.

Secondary symptoms: 1) Scanty menstrual flow and irregular menstruation; 2) Dark purple menstrual blood with clots, with temporary relief of pain after clot expulsion; 3) Breast distension and pain; 4) Chest tightness and discomfort;

Tongue appearance: Dark purple tongue with ecchymoses or petechiae, or purple stasis in the sublingual veins;

Pulse appearance: String-like and thready.

TCM Diagnosis: The primary symptom must be present, along with two or more secondary symptoms. Diagnosis is made based on the combination of tongue and pulse findings.

### 2.2 Inclusion and exclusion criteria

#### 2.2.1 Inclusion criteria

1. Meets the Western medicine, TCM diagnostic criteria, and TCM pattern differentiation criteria established for this study;
2. Female patients aged 15–45 years;
3. Regular menstrual cycle within 28 ± 7 days;
4. No prior treatment for this condition within the past month;
5. Voluntarily signed an informed consent form; patients under 18 years of age must have their legal guardian sign the informed consent form.

#### 2.2.2 Exclusion criteria

1. Patients with menstrual abdominal pain caused by pelvic organic diseases (e.g., endometriosis, adenomyosis, pelvic infection, etc.);
2. Patients allergic to the drugs used in this study;
3. Patients who have participated in other drug clinical trials within the past month and may interfere with this study;
4. Patients with severe cardiovascular, hepatic, renal, or haematological diseases;

#### 2.2.3 Exclusion and withdrawal criteria

1. Patients who develop sudden illnesses or experience physiological changes that affect the study and are unsuitable for continued treatment;
2. Failure to follow the study protocol, making it impossible to assess efficacy;
3. Self-administration of medications outside the study protocol, rendering efficacy assessment ineffective or inaccurate;
4. Poor compliance, refusal to cooperate with treatment, or voluntary withdrawal;
5. Pregnancy during treatment.

### 2.3 General information

#### 2.3.1 Sample size calculation

The sample size calculation adopts the formula:


$$n_{1}=n_{2}=\frac{1}{2}{\left(\frac{\mu_{\alpha }-\mu_{\beta }}{\sin^{-1}\sqrt{P_{1}}-\sin^{-1}\sqrt{P_{2}}}\right)}^{2}$$

The sample size was calculated based on the primary outcome of the total effective rate after three treatment cycles. The anticipated efficacy rate for the TCM group (P1 = 94%) was derived from prior studies by Professor Zhang Tingting, which demonstrated high efficacy (84%-94%) of the Stasis-Clearing and Meridian-Warming Method for dysmenorrhea (11, 12). We hypothesized that integrating this method with Cai’s foundational cyclical therapy would yield an efficacy at the higher end of this observed range. The estimated efficacy rate for the ibuprofen control group (P2 = 63%) was based on a recent clinical trial by Xu et al. (13), which specifically reported the efficacy of ibuprofen monotherapy (400 mg/day) for primary dysmenorrhea. Using these parameters (P1 = 0.94, P2 = 0.63, α=0.05, β=0.10, power=90%), a two-proportion power analysis was performed. The calculated sample size was 33 per group. To account for a potential 20% dropout rate, 40 participants per group (total N = 80) were recruited.

#### 2.3.2 Randomization and allocation concealment

A statistician not involved in patient recruitment or outcome assessment generated the allocation sequence using the random number generator function in SPSS software (version 25.0). A fixed seed value of 2,000,000 was used to ensure reproducibility. The sequence was created for 80 participants with a 1:1 allocation ratio into either the TCM group or the ibuprofen group. The resulting allocation list was concealed using sequentially numbered, opaque, sealed envelopes (SNOSE). Upon a participant’s enrollment and after the completion of baseline assessments, the treating clinician opened the next envelope in sequence to reveal the group assignment. This process ensured that the allocation was concealed until the moment of assignment, preventing selection bias.

Between February and July 2025, 80 patients diagnosed with Qi stagnation and blood stasis-type dysmenorrhoea were enrolled from the TCM Department outpatient clinic at Shanghai East Hospital. Patients were randomly assigned to two groups using a random number table, with 40 patients in each group. Patients in the TCM group were aged 16–45 years, with an average age of 30.8 ± 8.04 years. Patients in the ibuprofen group were aged 15–46 years, with an average age of 29.38 ± 7.84 years.

#### 2.3.3 Blinding

Given the nature of the interventions (herbal decoctions vs. commercial capsules), it was not feasible to blind the patients or the practitioners administering the treatments. Therefore, this study was conducted as an open-label (unblinded) randomized controlled trial. However, to minimize the risk of performance and detection bias, the following measures were implemented (1): The researcher responsible for collecting post-treatment outcome data (TCM syndrome scores and VAS scores during follow-up visits) was blinded to the group allocation of the participants; and (2) The data analyst was also kept blinded to the group codes until the primary statistical analysis was completed.

Beyond the blinding of data analysts and outcome assessors described above, we implemented the following specific clinical measures during patient interactions to further minimize performance and detection bias (1): to minimize performance bias arising from differential enthusiasm or communication styles between groups, we developed and implemented a standardized script for all patient interactions during enrollment and follow-up visits. For all participants, regardless of group assignment, researchers used the following unified guidance: “Both treatment protocols in this study are currently recognized as effective approaches for primary dysmenorrhea in clinical practice. This research aims to compare the relative strengths and characteristics of these two established treatments. At present, there is no evidence suggesting that one protocol is absolutely superior to the other; and (2) While treating physicians were necessarily unblinded to group allocation, we ensured that the personnel collecting VAS scores and follow-up data were fully blinded. The specific procedure was as follows: 1) a designated research assistant, who was not involved in treatment allocation or delivery, contacted patients by telephone at each follow-up timepoint; 2) this assistant had access only to patient identification information and scheduled follow-up dates, but no knowledge of group assignment; 3) the assistant used a standardized telephone script to guide patients through VAS self-assessment; 4) all VAS scores were recorded directly into a database with participant codes but no group identifiers; 5) group codes were only revealed to the statistician after completion of the primary analysis.

### 2.4 Treatment methods

#### 2.4.1 Basic intervention

Both groups of participants were advised to adopt a healthy lifestyle during the treatment period: maintaining emotional well-being, avoiding exposure to cold and wind, and avoiding raw or cold foods, etc.

#### 2.4.2 Ibuprofen group

Patients were administered ibuprofen sustained-release capsules 300 mg orally twice daily (morning and evening) for 2 consecutive days during the menstrual period, for a continuous treatment period of 3 menstrual cycles. This regimen provided a total daily dose of 600 mg (300 mg twice daily), consistent with the manufacturer’s prescribing information and standard clinical practice guidelines.

Ibuprofen sustained-release capsules specifications: 12 capsules per box, 0.3 g per capsule, manufactured by Shanghai Xinyi Tianping Pharmaceutical Co., Ltd., with batch number H31022720.

#### 2.4.3 Traditional Chinese medicine group

In accordance with CONSORT-CHM 2017 standards for individualized Chinese herbal medicine formulas (14), the following specific guidelines were established for modifying the core formulas based on individual patient presentations (**Supplementary Table 1**). All modifications were performed by licensed TCM practitioners with specialized training in Cai’s Gynecology.

Observation group: Based on the patient’s menstrual cycle, treatment with the modified Stasis-Clearing and Meridian-Warming Formula was administered during the premenstrual phase (3–5 days before menstruation) and during menstruation. (The formula consists of Chaihu, Zhike, Xiangfu, Hongteng, Puhuang, Wulingzhi, Yuanhu, Wuyao, Huixiang, Rougui, Chishao, Ruxiang, and Moyao, among others, etc.); During the postmenstrual phase (from the end of menstruation to ovulation) and the intermenstrual phase (from ovulation to 5 days before menstruation), patients were treated with a modified version of the Chaihushugan Decoction combined with the Siwu Decoction. (The formula consists of Chaihu, Zhike, Xiangfu, Hongteng, Gancao, Chenpi, Shudihuang, Danggui, Baishao, Chuanxiong, and Yuanhu, among others, etc.). The Chinese herbal medicines are prepared by the Chinese Medicine Department of Shanghai East Hospital, with one dose per day. Each dose is boiled to yield 200 ml of decoction, which is taken warm half an hour after breakfast and dinner. Treatment begins during the late phase of the first menstrual cycle after enrollment and continues for three menstrual cycles.

All herbal substances were authenticated by the Pharmacy Department of Shanghai East Hospital according to the Chinese Pharmacopoeia (2020 edition) standards (15). Voucher specimens for each batch were retained by the hospital pharmacy and are accessible upon request. The processing methods for key herbs followed traditional specifications: Yuanhu was vinegar-processed to enhance analgesic properties; Puhuang and Wulingzhi were wrapped during decoction to prevent irritation. Heavy metal and pesticide residue testing were conducted on each batch, with results confirming compliance with safety standards.

**Figure 1:**
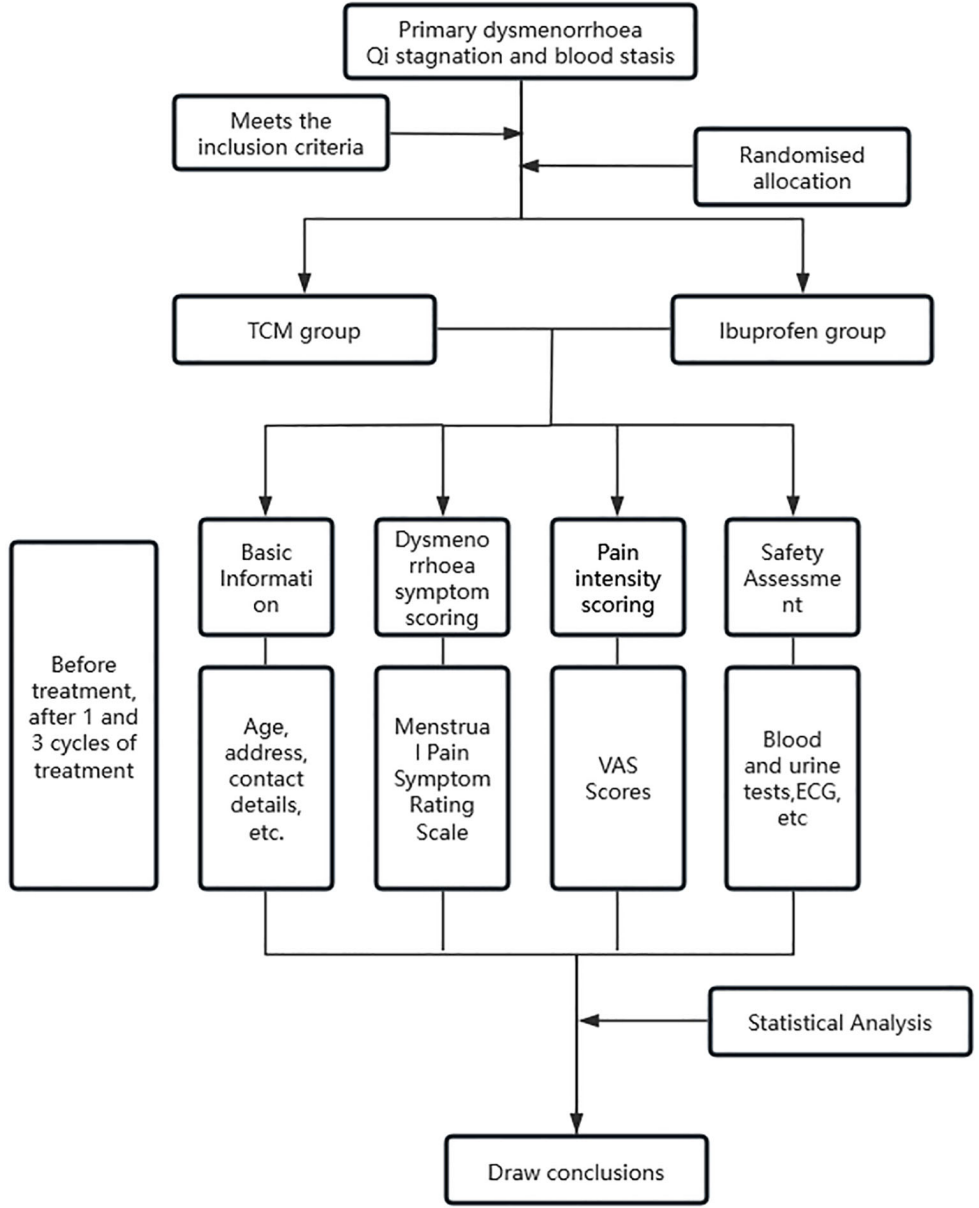
Flowchart of the integrated traditional chinese medicine (TCM) cyclical treatment protocol for primary dysmenorrhea.

The specific treatment schedule, illustrating the correspondence between the menstrual cycle phases and the two core formulas, is summarized in **Figure 1**.

#### 2.4.4 Ethics approval

This study was reviewed and approved by the Medical Ethics Committee of Shanghai East Hospital (Ethics Approval Number: 2025YS-046) and registered on the International Traditional Medicine Clinical Trials Registry Platform (Registration Number: ITMCTR2025000425).

### 2.5 Observation items and methods

#### 2.5.1 Dysmenorrhoea symptom scoring

Record dysmenorrhoea symptom scores before treatment, after one menstrual cycle of treatment, and after three menstrual cycles of treatment (refer to the ‘Guidelines for Clinical Research on New Traditional Chinese Medicines’) (16).

Evaluate the efficacy of dysmenorrhoea treatment according to the ‘Guidelines for Clinical Research on New Traditional Chinese Medicines for the Treatment of Dysmenorrhoea,’ with the following criteria (1): Cure: Score reduced to 0 points, with abdominal pain and associated symptoms completely resolved (2); Marked improvement: Score reduced by ≥50%, with significant relief of abdominal pain and improvement in other symptoms (3); Improvement: Score reduced by 25% to<50%, with relief of abdominal pain and improvement in other symptoms (4); No improvement: No significant improvement in symptoms after treatment, with score reduction<25%.

#### 2.5.2 Pain intensity scoring

The VAS scoring method was used: VAS scores were recorded before treatment, after 1 menstrual cycle of treatment, and after 3 menstrual cycles of treatment. A telephone follow-up VAS score was obtained 1 menstrual cycle after the patient completed treatment.

Specific procedure: The physician uses a 10 cm ruler as a tool, where 0 indicates no pain and 10 indicates unbearable pain. The patient marks the ruler according to the severity of their dysmenorrhoea pain, resulting in the corresponding score.

#### 2.5.3 Recurrence rate scoring

Follow-up for recurrence 1 menstrual cycle after completing treatment; if the VAS pain score worsens compared to the score 3 months after treatment, it is considered a recurrence; if the score remains the same or improves, it is considered non-recurrence.

### 2.6 Statistical methods

Statistical analysis was performed using SPSS 25.0 software. Continuous data are expressed as mean ± standard deviation (SD) For data following a normal distribution, independent samples t-tests were used for intergroup comparisons, and paired samples t-tests were used for intra-group comparisons before and after treatment. For data not following a normal distribution, non-parametric tests were used. Ordinal count data were analyzed using the rank sum test. All tests were two-sided, and P< 0.05 was considered statistically significant.

## 3 Results

### 3.1 Baseline data

A total of 80 patients were enrolled and randomized, with 40 assigned to each group. All participants completed the treatment and follow-up, resulting in no dropouts. As presented in **Table 1**, the two groups were comparable at baseline, with no statistically significant differences in age (P = 0.626), Dysmenorrhoea Symptom Scores (P = 0.135), or VAS pain scores (P = 0.885). This confirms that the randomization process was successful in creating equivalent groups for comparison.

**Table 1:** Comparison of two groups of baseline data (mean ± SD).

Group	n	Age	Dysmenorrhoea symptom scores	VAS scores
TCM	40	30.8 ± 8.04	12.84 ± 2.30	7.23 ± 1.38
Ibuprofen	40	29.38 ± 7.84	11.64 ± 2.69	6.23 ± 1.30
P		0.626^a^	0.135^b^	0.885^b^

^a^t-test. ^b^Mann–Whitney U test.

### 3.2 Pre-and post-intervention scores for TCM syndromes of dysmenorrhoea

The overall efficacy rate was identical in both groups at 97.5% after three treatment cycles (**Table 2**). However, the distribution of efficacy grades differed between the groups. A higher proportion of patients in the ibuprofen group achieved a “Cure” (57.5%) after three cycles compared to the TCM group (40.0%). Conversely, the TCM group had a higher number of patients in the “Marked improvement” (27.5%) and “Improvement” (30.0%) categories.

**Table 2:** Comparison of two groups of efficacy evaluation.

Group	n	Cured	Markedly effective	Effective	Ineffective	Overall efficacy rate (%)
TCM	40	16	11	12	1	97.5
Ibuprofen	40	23	10	6	1	97.5

The detailed results of the progressive improvement in TCM syndrome scores across treatment cycles are shown in **Table 3**. Both groups demonstrated significant within-group improvements in TCM syndrome scores after treatment. In the TCM group, scores showed a progressive and significant reduction from baseline to cycle 1 (P<0.01), and again from cycle 1 to cycle 3 (P<0.01). In contrast, the ibuprofen group showed a sharp reduction after the first cycle (P<0.01), but no further significant improvement from cycle 1 to cycle 3 (P = 0.844). Between-group comparisons revealed that the ibuprofen group was superior to the TCM group in reducing syndrome scores after the first cycle (P<0.01). However, this difference was no longer statistically significant by the end of the third cycle (P = 0.083), indicating that the TCM regimen achieved a comparable level of symptom control with prolonged treatment.

**Table 3:** Comparison of two groups of dysmenorrhoea symptom scores (mean ± SD, Mann–Whitney U test).

Dysmenorrhoea Symptom Scores	Before treatment	After 1 cycle of treatment	P (before treatment vs 1 cycle treatment)	After 3 cycles of treatment	P (1 cycle treatment vs 3 cycles treatment)	P (before treatment vs 3 cycles treatment)
TCM	12.84 ± 2.30	7.45 ± 4.21	< 0.01	3.91 ± 3.89	< 0.01	< 0.01
Ibuprofen	11.64 ± 2.69	3.10 ± 3.91	< 0.01	3.00 ± 3.83	0.844	< 0.01
P		< 0.01		0.083		

### 3.3 VAS scores before and after intervention

Both treatment regimens led to significant reductions in pain intensity, as measured by VAS (**Table 4**). Within-group analyses revealed distinct temporal patterns. In the TCM group, VAS scores decreased significantly from baseline after one treatment cycle (P<0.01) and showed further progressive improvement from cycle 1 to cycle 3 (P<0.01). Conversely, in the ibuprofen group, the pronounced pain relief achieved after the first cycle (P<0.01) was not sustained with additional improvement, with no significant change between cycle 1 and cycle 3 (P = 0.795). Between-group comparisons of VAS scores demonstrated that the ibuprofen group provided superior pain relief after the first cycle (P<0.01). However, this initial advantage diminished over time. After three treatment cycles, the difference in VAS scores between the two groups was no longer statistically significant (P = 0.059), indicating that the TCM regimen eventually achieved a comparable level of pain control.

**Table 4:** Comparison of two groups of VAS scores (mean ± SD, Mann–Whitney U test).

VAS Scores	Before treatment	After 1 cycle of treatment	P (before treatment vs 1 cycle treatment)	After 3 cycles of treatment	P (1 cycle treatment vs 3 cycles treatment)	P (before treatment vs 3 cycles treatment)
TCM	7.23 ± 1.37	3.9 ± 2.51	< 0.01	1.6 ± 1.81	< 0.01	< 0.01
Ibuprofen	6.23 ± 1.30	0.78 ± 1.05	< 0.01	0.73 ± 1.04	0.795	< 0.01
P		< 0.01		0.059		

### 3.4 Recurrence follow-up one menstrual cycle after completion of treatment

All patients in both groups were successfully followed up. The recurrence rate was significantly lower in the TCM group (5 out of 39 effectively treated patients, 12.8%) compared to the ibuprofen group (35 out of 39 effectively treated patients, 89.7%).

### 3.5 Safety evaluation

No adverse events occurred in either group during the treatment period.

## 4 Discussion

This study demonstrates that both treatments effectively alleviated pain after one cycle, with ibuprofen providing superior short-term relief. However, after three cycles, the TCM regimen achieved comparable efficacy to ibuprofen, while also demonstrating a progressive improvement across treatment cycles. This indicates that ibuprofen treatment for dysmenorrhoea can achieve the highest efficacy immediately in the short term, while Cai’s Gynaecological Cycle Therapy combined with the Stasis-Clearing and Meridian-Warming Therapy also showed significant short-term efficacy, but it was weaker than oral ibuprofen. With continued treatment, the effects gradually strengthened, and after three menstrual cycles, the efficacy reached the same level as ibuprofen. The most clinically significant finding of this trial is the markedly lower recurrence rate observed in the TCM group (12.8%) compared to the ibuprofen group (89.7%) during the single menstrual cycle followed after treatment. This suggests that the TCM protocol may have carry-over effects that sustain its benefits immediately after the end of active treatment, whereas the effect of ibuprofen is strictly contingent on its administration.

TCM treatments such as herbal medicine, acupuncture, acupoint application, and massage are widely used for primary dysmenorrhoea (17). This study builds upon the foundation of established TCM therapies by innovatively integrating the academic expertise of two successive heirs of the Cai’s Gynecology school, a Shanghai intangible cultural heritage, and integrates and innovates upon the academic experiences of the seventh-generation successor, Professor Cai Xiaosun, and the eighth-generation successor, Professor Zhang Tingting, with the aim of exploring better treatment methods. The Cai Family Gynaecology pioneered the TCM Cycle Therapy. In the early 1970s, Professor Cai Xiaosun proposed the four physiological characteristics of the menstrual cycle and the corresponding treatment strategies. The menstrual cycle is divided into four phases: the menstrual phase (from the onset of menstruation to its cessation), the post-menstrual phase (from cessation to ovulation), the intermenstrual phase (ovulation), and the pre-menstrual phase (from ovulation to the onset of the next menstruation). However, in clinical practice, patients cannot visit the clinic weekly, which may lead to poor compliance. This study adjusted the medication schedule according to the menstrual cycle, using two formulas for treatment. Patients only need to visit the clinic at least twice a month, reducing the number of visits and improving compliance. This may also be one of the reasons why no patients dropped out of this study.

Cai’s gynaecological treatment for dysmenorrhoea emphasizes addressing the root cause as the primary goal, with pain relief as a secondary objective. Dysmenorrhoea has numerous causes in TCM, including cold stagnation and blood stasis, qi stagnation and blood stasis, damp-heat accumulation, qi and blood deficiency, and liver and kidney deficiency. Cai’s gynaecology holds that although the causes of dysmenorrhoea are numerous, the fundamental issue lies in the obstruction of menstrual blood flow, leading to stasis and pain. Therefore, the primary treatment method is to promote ‘flow.’.

The liver stores blood and governs the smooth flow of qi, preferring a harmonious and balanced state. It is particularly closely related to a woman’s menstruation and pregnancy, hence the saying, ‘The liver is the innate organ of women.’ As the saying goes, ‘To treat blood disorders, one must also regulate qi,’ and ‘To regulate menstruation, one must first regulate qi.’ We administer the Chai Hu Shu Gan San combined with the Si Wu Tang formula with appropriate modifications during the postmenstrual period and intermenstrual period of the patient. (The formula consists of Chai Hu, Zhi Ke, Xiang Fu, Hong Teng, Gan Cao, Chen Pi, Shu Di, Dang Gui, Bai Shao, Chuan Xiong, Yuan Hu, etc.) The focus is on regulating the liver, with an emphasis on harmonizing qi and blood.

Patients with qi stagnation and blood stasis-type dysmenorrhoea often experience emotional distress, liver dysfunction, stagnation of qi and blood in the Chong and Ren meridians, and impaired menstrual flow, leading to the principle of ‘no flow, no pain.’ We treat such cases with Chaihushugan Decoction combined with the Siwu Decoction, plus Hongteng and Yuanhu. Chaihushugan Decoction primarily functions to regulate the liver, relieve depression, promote qi circulation, and alleviate pain. In the formula, Chaihu serves as the principal herb to regulate the liver and relieve depression, while Baishao softens the liver and alleviates urgency, and Zhike promotes qi circulation and broadens the chest as auxiliary herbs. These are supplemented by Xiangfu to regulate menstruation and alleviate pain, Chenpi to regulate qi and strengthen the spleen, Chuanxiong to activate blood circulation and promote qi circulation, and Gancao to harmonize the actions of all herbs. The entire formula emphasizes the harmonious regulation of qi and blood, as well as the concurrent treatment of the liver and spleen. Chaihushugan Decoction has been used clinically for the treatment of liver qi stagnation syndrome for nearly 400 years. Its efficacy in improving mood and alleviating pain through mechanisms such as inhibiting inflammatory factors and regulating neurotransmitter release has been confirmed by network pharmacology, experimental, and clinical studies (18, 19).

The combination of Chaihushugan San and Siwu Decoction was used to address the root cause by soothing the liver and harmonizing blood. Modern studies suggest this formula may modulate inflammatory factors and neurotransmitters (18, 19), while Siwu Decoction has been shown to relax uterine smooth muscle (20, 21). The addition of Yuanhu and Hongteng enhances the effects of promoting blood circulation, regulating qi, and relieving pain. Yuanhu has ample clinical evidence to support its analgesic mechanism and is one of the most commonly used analgesic drugs in clinical practice (22, 23). The use of Hongteng originates from Professor Dai Deying, a renowned TCM practitioner in Shanghai, who achieved remarkable results in treating dysmenorrhoea with the Hongteng formula (24). Here, in addition to its blood-activating and pain-relieving effects, it also helps prevent the warming effects of the Si Wu Decoction. Of course, Hongteng also has extensive modern medical theoretical support for its effects (25).

According to Cai’s Gynaecology, treatment for regulating menstruation and relieving pain should begin three days before menstruation, hence the Stasis-Clearing and Meridian-Warming Therapy is administered during the premenstrual and menstrual periods with appropriate adjustments. The Stasis-Clearing and Meridian-Warming Therapy is a summary of the clinical experience of Professor Zhang Tingting, a renowned TCM practitioner in Shanghai. Professor Zhang emphasizes the impact of cold pathogens on female physiology and uses this formula to treat dysmenorrhoea and chronic pelvic pain in patients with endometriosis and adenomyosis, while also reducing the recurrence rate after surgery (11, 26). In the formula, Chishao and Wuyao serve as the primary herbs, leveraging their effects of promoting blood circulation, removing blood stasis, warming the meridians, and alleviating pain. Puhuang and Wu Ling Zhi are components of the Shi Xiao San formula, traditionally regarded as essential herbs for treating pain caused by blood stasis. They act on multiple targets related to inflammation and neural pathways, exhibiting analgesic effects, improving uterine artery blood flow, inhibiting thrombus formation, and reducing inflammatory responses (27, 28); HuiXiang and Rougui assist Wuyao in promoting blood circulation, warming the meridians, and alleviating pain. Their pungent nature disperses and their warming nature relaxes, enabling the entire formula to act without stagnation and reach the affected area. Additionally, there are examples of using Huixiang alone to treat primary dysmenorrhoea with efficacy (29). Furthermore, considering the characteristics of patients with primary dysmenorrhoea due to qi stagnation and blood stasis, and adapting to the physiological characteristics of patients before menstruation, the formula retains herbs such as Chaihu, Zhike, Xiangfu, Hongteng, and Yuanyu for liver-soothing, qi-regulating, and pain-relieving effects, while adding the herb pair of Ruxiang and Moyao to enhance qi-regulating and blood-stasis-resolving effects. The entire formula combines liver-soothing and qi-regulating with warming the meridians and resolving stasis. The formula uses warming, harmonizing, and clearing herbs together to achieve the effects of clearing stasis, warming and unblocking, liver-soothing, and pain relief.

In the control group, we used oral ibuprofen for treatment. Ibuprofen is a commonly used non-steroidal anti-inflammatory drug (NSAID) for the treatment of dysmenorrhoea in clinical practice (1, 30). It inhibits prostaglandin synthase, thereby blocking prostaglandin synthesis and alleviating pain (31). In our study, we found that ibuprofen treatment was indeed highly effective in treating primary dysmenorrhoea. During the first treatment cycle, patients’ pain symptoms improved promptly. Of course, the efficacy of this drug has already been widely recognized. However, the efficacy of ibuprofen in treating dysmenorrhoea is not sustained. Once the active components are metabolized in the body, the analgesic effect ceases abruptly. Therefore, in the follow-up of patients after the conclusion of this study, the majority of patients who ceased taking ibuprofen still experienced abdominal pain, and the severity of the pain did not diminish compared to before. Among patients who regularly took TCM and achieved effective treatment, only 5 patients reported more severe pain than the previous menstrual cycle during telephone follow-up after completing the treatment course.

The most striking finding of this trial is the dramatically lower recurrence rate in the TCM group (12.8%) compared to the ibuprofen group (89.7%). This underscores a fundamental distinction between a symptomatic and a root-cause approach. While NSAIDs like ibuprofen provide excellent ‘rescue’ analgesia by blocking prostaglandin production, their effect is transient (29). The high recurrence rate with ibuprofen is mechanistically expected: as a non-steroidal anti-inflammatory drug, ibuprofen acts by inhibiting cyclooxygenase enzymes, thereby reducing prostaglandin synthesis and providing temporary pain relief during the current menstrual cycle (32). However, NSAIDs do not address the underlying pathophysiology of dysmenorrhea, nor do they modify the disease process. Consequently, upon drug discontinuation, prostaglandin production resumes, and pain typically recurs with the next ovulatory cycle. The 89.7% recurrence rate observed in our study—occurring within the very first menstrual cycle after treatment completion—is actually higher than some previous reports. This may be explained by (1): our stricter definition of recurrence (any worsening of VAS compared to post-treatment score) (2); the very short follow-up window (one cycle) which captured immediate recurrence that might otherwise be missed in longer-interval follow-ups; and (3) our study population’s specific TCM pattern (Qi stagnation and blood stasis), which may represent a subgroup particularly prone to rapid recurrence after NSAID withdrawal. Our results suggest that the TCM protocol, by systematically regulating the menstrual cycle across its phases, may induce longer-lasting physiological changes. This finding aligns with the growing international focus on ‘disease modification’ in chronic pain conditions, rather than mere palliation (33). For instance, a recent systematic review by Yu et al. (2025) on acupuncture for dysmenorrhea also reported sustained effects post-treatment, suggesting that complex TCM interventions can indeed alter the underlying disease process (34). While our herbal protocol is different, the principle of achieving lasting benefit beyond the treatment period is congruent and points to a promising area for future mechanistic research.

It is important to consider whether the observed differences between groups might be attributable, at least in part, to differential expectancy effects arising from the more intensive TCM treatment regimen. While we cannot entirely exclude this possibility, several factors suggest that expectancy alone is unlikely to explain our findings. First, the recurrence rate difference—the most clinically significant outcome—was assessed after treatment completion, when the immediate context of therapeutic encounters had been removed. Second, the progressive improvement pattern in the TCM group across three cycles (P< 0.01 for both outcomes) contrasts with the stable, non-progressive pattern in the ibuprofen group; pure expectancy effects would typically manifest as immediate improvement that may plateau but would not necessarily show cumulative enhancement across cycles. Third, the objective nature of recurrence is less vulnerable to reporting bias than contemporaneous pain assessment during active treatment. Nevertheless, we acknowledge this as a limitation and recommend that future studies incorporate formal expectancy measurement using validated instruments to quantify and statistically adjust for these important non-specific factors.

## 5 Limitations

This study has several limitations that should be considered when interpreting the results. The lack of laboratory indicators (e.g., prostaglandin PGF2α or inflammatory cytokine levels) means we cannot objectively quantify the biological mechanisms underlying the observed clinical improvements. The unblinded design introduces the potential for performance and detection bias, as both patients and clinicians were aware of the treatment allocation, which may have influenced subjective reporting of pain (VAS scores). The follow-up period of only one menstrual cycle post-treatment was primarily constrained by the study timeline and available funding. This single-cycle follow-up, while sufficient to detect immediate post-treatment recurrence, does not provide comprehensive evidence of long-term therapeutic stability. This is particularly important for a chronic condition such as primary dysmenorrhea, where patients and clinicians seek solutions that provide sustained benefit over many months or years. The sample size, while calculated *a priori*, was relatively small, which may limit the generalizability of our findings and the power to detect smaller, yet potentially clinically significant, differences between the groups. This study employed an active-controlled design, comparing the TCM protocol against ibuprofen, a first-line conventional therapy for dysmenorrhea. While a placebo control would have been valuable to isolate the specific effect of the TCM intervention from the natural history of the condition and placebo effects, we deemed an active comparator to be more clinically relevant and ethical for this patient population. We acknowledge that without a placebo arm, we cannot fully quantify the absolute magnitude of the specific therapeutic effect attributable to the TCM intervention beyond contextual effects. The lack of objective markers such as prostaglandin PGF2α levels, uterine artery blood flow indices, or inflammatory cytokine measurements limits our ability to understand the physiological basis of the “root-cause” treatment effect. While we have provided detailed guidelines for formula modifications in accordance with CONSORT-CHM 2017 standards, we acknowledge that some degree of clinical judgment remains inherent in individualized TCM practice. The modifications described represent standardized decision rules developed by the research team to balance ecological validity (reflecting real-world clinical practice) with reproducibility. Future pragmatic trials may benefit from further operationalizing these criteria through algorithmic decision support tools. Finally, we acknowledge the absence of formal measurement and statistical adjustment for expectancy effects. the more intensive 3-month TCM regimen may have generated greater non-specific therapeutic effects—including patient expectations, attention from healthcare providers, and the ritual of treatment—compared to the simpler ibuprofen regimen (two days of oral capsules per cycle). These factors could theoretically contribute to the observed between-group differences, particularly for subjective outcomes such as VAS pain scores. Ideally, we would have measured baseline treatment expectancy using validated instruments, and included expectancy scores as covariates in the analysis to isolate the specific treatment effect beyond non-specific factors. However, this study was designed and initiated prior to the routine incorporation of expectancy measurement in TCM trials, and we did not collect these data.

## 6 Conclusion

In conclusion, this randomized controlled trial confirms that Cai’s Gynaecological Cycle Therapy combined with the Stasis-Clearing and Meridian-Warming Method is an effective treatment for primary dysmenorrhea of the Qi stagnation and blood stasis type. The study’s key contributions are threefold (1): it validates a phase-specific TCM protocol that systematically addresses the menstrual cycle, achieving progressive improvement across three cycles (2); it demonstrates that while ibuprofen provides superior short-term relief after one cycle, the TCM regimen achieves comparable efficacy by cycle 3; and (3) most importantly, it reveals a dramatically lower one-month post-treatment recurrence rate in the TCM group compared to the ibuprofen group, consistent with the known mechanism of NSAIDs which provide temporary analgesia without disease-modifying effects. These findings have important clinical implications. The TCM protocol offers a viable alternative to NSAID monotherapy for patients seeking sustained benefits beyond immediate pain relief. The low recurrence rate suggests that this approach may modify the underlying disease process rather than merely suppressing symptoms—a particularly valuable characteristic for a chronic, recurrent condition that affects quality of life and productivity in young women. For clinicians, this protocol provides a structured, phase-specific treatment framework that can be implemented in practice with the standardized modification guidelines provided in this manuscript. Future research priorities include (1): extending the follow-up duration to assess long-term outcomes (2); conducting multi-center trials incorporating objective biomarkers (3); elucidating the underlying mechanisms through basic science investigations; and (4) exploring implementation strategies to facilitate integration of this approach into broader healthcare settings.

## Data Availability

The raw data supporting the conclusions of this article will be made available by the authors, without undue reservation.
