# Supplementary material for: Cai’s gynecology cyclical therapy with stasis-clearing and meridian-warming method for primary dysmenorrhea: a randomized controlled trial
Source: Front Endocrinol (Lausanne). 2026 May 14;17:1752657. doi: 10.3389/fendo.2026.1752657 (PMC13215811; doi:10.3389/fendo.2026.1752657)
Supplement: Supplementary file 1 [file Table1.docx]

Table S1. Standardized Modification Guidelines for Herbal Formulas

| Symptom presentation | Modification strategy | Dosage adjustment range | Rationale |
| --- | --- | --- | --- |
| Severe pain (VAS > 7) | Increase Yuanhu (Corydalis Rhizoma) | 10g → 15g | Yuanhu contains alkaloids (tetrahydropalmatine) with dose-dependent analgesic effects; higher dosage within safe range enhances pain relief |
| Severe pain with large clots | Increase Puhuang (Typhae Pollen) and Wulingzhi (Trogopterori Faeces) | 10g → 12-15g each | Both herbs synergistically activate blood circulation and resolve stasis; dosage increase enhances antiplatelet aggregation and fibrinolytic effects |
| Cold sensation in lower abdomen | Increase Huixiang (Foeniculi Fructus) and Rougui (Cinnamomi Cortex) | Huixiang: 6g → 10g; Rougui: 3g → 5g | Warming herbs dispel cold and unblock meridians; dosage increase indicated when cold-phlegm stagnation is prominent |
| Severe breast distension and emotional irritability | Increase Chaihu (Bupleuri Radix) and Xiangfu (Cyperi Rhizoma) | 10g → 12-15g each | Enhanced liver-soothing and qi-regulating effects for pronounced premenstrual symptoms |
| Younger age (15-25 years) | Standard or lower dosage range | Use lower end of therapeutic range | Younger patients typically more sensitive to herbal actions and may require less aggressive dosing |
| Older age (36-45 years) | Standard or moderately increased dosage | May use upper end of range | Older patients may have more chronic stasis patterns requiring stronger intervention |
| Mild symptom severity | No modification or minimal adjustment | Use standard dosage | Mild presentations respond adequately to base formula without augmentation |
| Moderate-severe symptom severity | Targeted increases in 1-3 key herbs | As specified above | Multi-symptom presentations require strategic enhancement of specific therapeutic actions |
