# Supplementary material for: Cai’s gynecology cyclical therapy with stasis-clearing and meridian-warming method for primary dysmenorrhea: a randomized controlled trial
Source: Front Endocrinol (Lausanne). 2026 May 14;17:1752657. doi: 10.3389/fendo.2026.1752657 (PMC13215811; doi:10.3389/fendo.2026.1752657)
Supplement: Supplementary file 2 [file DataSheet1.doc]

**CONSORT 2010 Flow Diagram**

**Allocation**

**Analysis**

**Follow-Up**

**Enrollment**

Assessed for eligibility (n=92 )

Excluded (n=12 )

♦  Not meeting inclusion criteria (n=4 )

♦  Declined to participate (n=8 )

♦  Other reasons (n=0 )

Analysed (n=40 )
♦ Excluded from analysis (give reasons) (n=0 )

Lost to follow-up (give reasons) (n=0 )

Discontinued intervention (give reasons) (n=0 )

Allocated to intervention (n=40 )

♦ Received allocated intervention (n=40 )

♦ Did not receive allocated intervention (give reasons) (n=0 )

Lost to follow-up (give reasons) (n=0 )

Discontinued intervention (pregnancy) (n=0 )

Allocated to intervention (n=40 )

♦ Received allocated intervention (n=40 )

♦ Did not receive allocated intervention (give reasons) (n=0 )

Analysed (n=40 )
♦ Excluded from analysis (give reasons) (n=0 )

Randomized (n=80 )
